# Specific zinc finger‐induced methylation of PD‐L1 promoter inhibits its expression

**DOI:** 10.1002/2211-5463.12568

**Published:** 2019-05-14

**Authors:** Xue Li, Zhenni Wang, Jiansheng Huang, Huazao Luo, Sibo Zhu, Han Yi, Liuhai Zheng, Bo Hu, Lili Yu, Lingzhi Li, Jun Xie, Naishuo Zhu

**Affiliations:** ^1^ Laboratory of Molecular Immunology State Key Laboratory of Genetic Engineering School of Life Sciences Institute of Biomedical Sciences Fudan University Shanghai China; ^2^ Institutes of Biomedical Science Fudan University Shanghai China

**Keywords:** DNA methylation, PD‐L1, tumor, zinc finger

## Abstract

DNA methylation of promoter regions is often associated with epigenetic silencing of gene expression, and DNA methyltransferase (DNMTs) has been used to suppress gene expression. In order to explore the synergistic roles of two methyltransferase members Dnmt3a and Dnmt1, we constructed expression plasmid that could express a recombinant DNMTs consisting of the C‐terminal domains of both Dnmt3a and Dnmt1 fused to a zinc finger domain which binds to the PD‐L1 promoter of human prostate cancer cells (DU145). Programmed death ligand 1 (PD‐L1, B7‐H1, CD‐274) is a transmembrane protein widely expressed on antigen‐presenting and other immune cells. The interaction of PD‐L1 with its receptor PD‐1 is considered an ‘immune checkpoint' for possible cancer therapy. DU145 cells treated with the Dnmt3aC‐1C plasmid showed significantly reduced expression of PD‐L1 as compared to Dnmt3aC or Dnmt1C alone. Our results show that the fusion of Dnmt1 improves the methylation activity of Dnmt3a and enhances its biological functions. This combinatorial strategy can be used to better control PD‐L1 expression to support cytotoxic T lymphocytes (CTL) response against tumors.

**Abbreviations**
CTL: cytotoxic T lymphocytes
DNMTs: DNA methyltransferase
MSP: methylation‐specific PCR

Programmed cell death ligand 1 (PD‐L1, B7‐H1, CD274) is a transmembrane protein that is widely expressed on antigen‐presenting and other immune cells, and is unregulated on tumor cells from a broad range of cancer types. Binding of PD‐L1 with its receptor PD‐1 on T cells delivers a signal that inhibits TCR‐mediated activation of IL‐2 production and T‐cell proliferation. PD‐L1 binding to PD‐1 also contributes to ligand‐induced TCR downregulation during antigen presentation to naive T cells 1. In addition, the PD‐1/PD‐L1 interaction is also implicated in autoimmunity 2, 3.

Using a PD‐L1 inhibitor that blocks the interaction of PD‐L1 with the PD‐1 receptor can prevent cancer from evading host immune system. So, blocking the PD‐1/PD‐L1 interaction can suppress tumor immune escape, thereby improving the ability of initial T‐cell activation and CTL killing activity 4. Several PD‐L1 inhibitor antibodies are being trialed for use in advanced melanoma, non‐small‐cell lung cancer, renal cell carcinoma, and bladder cancer 5. Immunotherapy with these immune checkpoint inhibitors appears to shrink tumors in a greater number of patients across a wider range of tumor types. Hence, PD‐L1 inhibitors are considered to be the most promising drug category for many different cancers 6. However, the large size of the antibody limits its ability to properly penetrate solid tumors in order for it to act. Shrinking the tumor with other treatment modalities before using immune checkpoint immunotherapy could avoid this 7, 8.

DNA methylation of promoter regions is often associated with epigenetic silencing 9. In normal tissue, around 80% of CpGs are methylated whereas CpG islands in promoter regions of active genes are hypomethylated 10. In cancer, there is a shift in the pattern of DNA methylation toward a global hypomethylation, whereas certain CpGs, often in promoter regions of tumor suppressor genes, are thought to become hypermethylated 10, 11. Nevertheless, recent publications show that the aberrant DNA methylation, especially in cancer, is much more complex 9. Methylation in promoter regions correlates negatively with gene expression and makes important genes such as tumor suppressor genes, DNA repair, and other genes lose their functions 12.

The DNA methyltransferase (DNMTs) comprises a family of DNA‐modifying enzymes that have a central role in epigenetic gene regulation. Global cytosine methylation patterns in mammals appear to be established by a complex interplay of at least three independently encoded DNMTs: DNMT1, DNMT3A, and DNMT3B 13. The primary function of Dnmt1 is the maintenance of the DNA methylation pattern during replication, whereas Dnmt3a and Dnmt3b function as *de novo* methyltransferases 14. The human DNMTs 1, 3A, and 3B coordinate mRNA expression in normal tissues and overexpression in tumors. The expression levels of these DNMTs are reportedly elevated in cancers of the colon, prostate, breast, liver, and in leukemia. Conversely, reduction in DNMT1 levels appears to have protective effects 15.

DNA methyltransferases have been used to repress target gene through specific DNA methylation, with guidance to the promoter region of a target gene by fusing it to a specific zinc finger domain 16 . In this study, we explored the synergistic methylation activities of Dnmt3a and Dnmt1 on PD‐L1 gene promoter. We used a recombinant Ad‐ZF‐Dnmt3aC‐1C expression construct to inhibit the expression of PD‐L1 and thus prevent PD‐1/PD‐L1 interaction. This construct expresses a fusion protein that consists of the C‐terminal domains of both Dnmt3a and Dnmt1 fused with a zinc finger domain that could specifically bind to PD‐L1 promoter. Human prostate cancer cells (DU145) treated with Ad‐ZF‐Dnmt3aC‐1C showed significant reduction in PD‐L1 expression as compared to Ad‐ZF‐Dnmt3aC or Ad‐ZF‐Dnmt1C alone. Our results show that the fusion of Dnmt1C strengthens the biological functions of Dnmt3a. This strategy can be used to target genes of interest more efficiently, for example, to block PD‐1/PD‐L1 interaction thereby augmenting CTL activity for killing tumors.

## Materials and methods

### Study design

**Figure 1:**
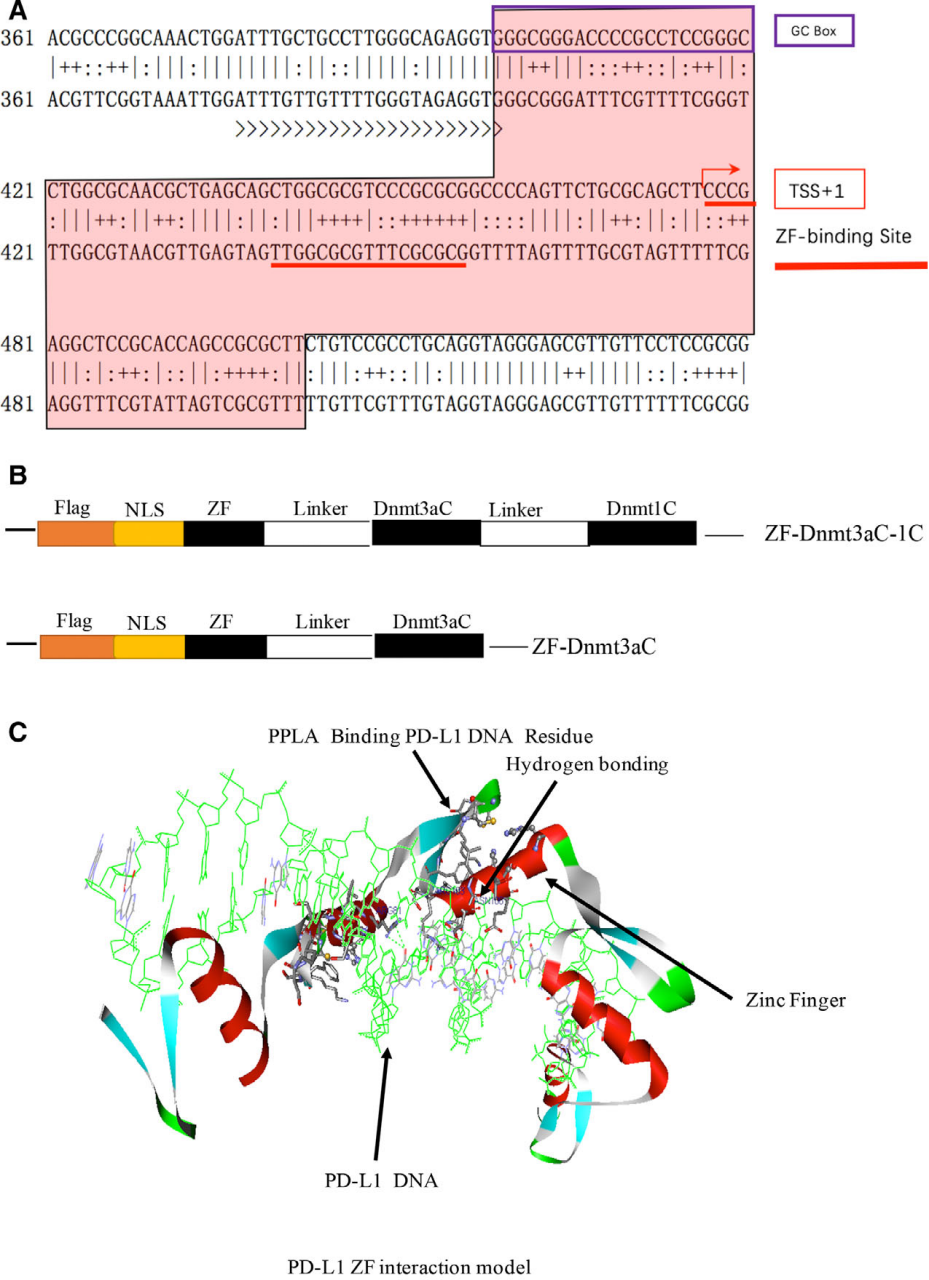
Zinc finger‐fused recombinant Dnmts vectors. (A) Putative methylation and zinc finger protein binding sites of PD‐L1 promoter. The second strand represents the sequence of the bisulfite converted methylated DNA (B) Schematic representation of zinc finger‐fused Dnmts vectors. (C) PD‐L1 ZF interaction model. The model is based on a crystal structure of zinc finger protein (PDB: 2I13).

We used Eukaryotic Gene Promoter Database to analyze PD‐L1 gene promoter sequence. CpG Island Analysis Software (http://www.urogene.org/methprimer) was used to analyze the core promoter sequences of potential methylation site (CpG island), and zinc finger protein binding site on PD‐L1 gene promoter was identified by the software (http://www.scripps.edu/barbas/zfdesign/register.php?query). Putative methylation and zinc finger protein binding sites on PD‐L1 promoter are shown in Fig. 1A.

### Recombinant adenovirus vector constructs

All the zinc finger‐fused Dnmts sequences were commercially inserted into replication‐deficient adenoviral vectors pDOV‐mCMV‐GFP‐3FLAG (Obio Technology, Shanghai, China) by Neuron Biotech, Shanghai, China, and abbreviated as Ad‐ZF‐Dnmt3aC‐1C.

The C terminus of Dnmt3a (2217–3074; GenBank: NM_175629.1) and Dnmt1 (3270–5028; GenBank: NM_001379.2) were generated from pcDNA3.1/Flag‐Dnmt3a and pcDNA3.1/Flag‐Dnmt1 by PCR using primers listed in Table 1. The Dnmt1C fragment was fused to the C terminus of Dnmt3aC (pZF‐Dnmt3aC) with a flexible linker (Gly4Ser)_2_ to generate pZF‐Dnmt3aC‐1C and this to the C terminus of zinc finger (ZF). These recombinant Dnmt constructs were commercially packed into replication‐defective adenoviral vector pDOV‐mCMV‐GFP‐3FLAG (Neuron Biotech) to generate Ad‐ZF‐Dnmt3aC‐1C. The pDOV‐mCMV‐GFP‐3FLAG was considered as negative control (Ad‐control). The resulting vectors are schematically represented in Fig. 1B.

**Table 1:** List of primers

Primer	Sequence
Dnmt3aC F	CCAGCTGAGAAGAGGAAG
Dnmt3aC R	CACACACGCAAAATACTCC
Dnmt1C F	AAAATCCGGGTCAACAAGT
Dnmt1C R	CTAGTCCTTAGCAGCTTC
IL‐4R promoter left M primer	TTATTTGGAAATTTAGTTGGGAGTC
IL‐4R promoter right M primer	AACGTAAAAAAAACCAAAAAACG
IL‐4R promoter left U primer	TATTTGGAAATTTAGTTGGGAGTTG
IL‐4R promoter right U primer	AACATAAAAAAAACCAAAAAACACT
Flag‐XPZF F	ATGGATTACAAGGATGACGACG
Flag‐XPZF R	GCTGGTTTTTTTGCCGGT
GAPDH F	CAAGGTCATCCATGACAACTTTG
GAPDH R	GTCCACCACCCTGTTGCTGTAG
PD‐L1 F	GTGGCATCCAAGATACAAACTCAA
PD‐L1 R	TCCTTCCTCTTGTCACGCTCA

### Cell culture and cells transduction

Human prostate cancer cells (DU145) were obtained from the American Type Culture Collection (ATCC, Rockville, MD, USA). Cells were cultured in standard DMEM with 10% fetal bovine serum, 1% penicillin/streptomycin at 37 °C, and 5% CO_2_. For transduction, 2.0 × 10^5 ^cells were cultured in 12‐well plates in regular growth medium until they reached 70–80% confluency. Cells were infected with Dnmts vectors at 80–100 MOIs (multiplicity of infection) per cell.

### Western blot analysis

Total proteins were collected from the infected cells after 24, 48, and 72 h, run on 10% SDS/PAGE, and detected with 3000× diluted anti‐PD‐L1 monoclonal antibody (Clone EPR1161, Abcam, Cambridge, UK) overnight. The antibody was removed, and the poly(vinylidene difluoride) membrane was washed three times for 5 min each in TBST. Anti‐rabbit secondary antibody (Abcam) was used at a dilution of 1 : 5000 in TBST and incubated for 1 h at room temperature. The antigen–antibody complexes were detected using the G:BOX F3 gel doc system (Syngene, Cambridge, UK).

### Flow cytometry

Cells were cultured in 6‐well plates in regular growth media until they reached 70–80% confluency. Cells were fixed with 4% PFA and washed. PD‐L1 was detected using PE‐conjugated anti‐PD‐L1 antibody (BD Biosciences, Franklin Lakes, NJ, USA) on Calibur flow cytometer (BD Bioscience), and data were analyzed using flowjo software (Tree Star, San Carlos, CA, USA).

### RNA preparation and quantitative real‐time PCR (qRT–PCR)

Total RNA from treated and control cells were isolated after 24, 48, and 72 h using Trizol reagent (Invitrogen, Carlsbad, CA, USA) according to the manufacturer's instructions. For each sample, 2 μg of total RNA was reversely transcribed using the QuantiTect Reverse Transcription Kit (Qiagen Inc., Valencia, CA, USA). Gene expression was determined using the DyNAmo Flash SYBR Green qPCR Kit (Thermo Scientific Inc., Foster, CA, USA) on the Stratagene Thermocycler (Mx3000). The primers used are listed in Table 1.

### Methylation‐specific PCR (MSP) assay and DNA extraction

To check the degree of methylation of PD‐L1 promoter, methylation‐specific PCR (MSP) was performed as described earlier 13. DU145 cells were cotransfected with plasmid Ad‐ZF‐Dnmt3aC, Ad‐ZF‐Dnmt1C, Ad‐ZF‐Dnmt3aC‐1C, or pDOV‐mCMV‐GFP‐3FLAG (Ad‐control) for 40 h. The promoter of interleukin‐4 receptor (IL‐4R) was also analyzed as off‐target control 14. Bisulfite reaction was performed on 500 ng of DNA. Sodium bisulfite conversion and purification of DNA were performed with the EpiTech Plus Bisulfite Conversion Kit (Qiagen, Hilden, Germany). Cell genomes were extracted, and a fragment corresponding to the PD‐L1 promoter sequence was amplified with primers designed with the methprimer software (Beijing, China) for methylated DNA (Table 1). The amplified fragment was cloned into the pMD19‐T vector and sequenced. Data analysis and data quality check were carried out using the biq analyzer software, a standard tool for processing DNA methylation data from bisulfite sequencing (http://biqanalyzer.bioinf.mpi-inf.mpg.de/).

### Statistical analyses

Statistical tests were performed with the spss software (SPSS Inc., Chicago, IL, USA). The data are reported as means ± SD. Different experimental groups were compared by Student's *t‐test*. A value of *P *<* *0.05 was considered statistically significant.

## Results

Analysis with Eukaryotic Gene Promoter Database showed that PD‐L1 gene promoter sequence is highly methylated in normal cells and is hypomethylated in tumor cells. Using Tools Ver3.0 design software, we identified PD‐L1 gene promoter and GC box including downstream GTCCGCCTGCAGGTAGGGAGC zinc finger binding site. The 100‐bp‐long fragment contained 15 potential methylation sites, of which three were located in GC box (Fig. 1A).

### Recombinant Dnmts protein expression

**Figure 2:**
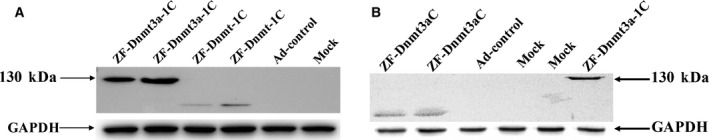
Expression of recombinant ZF‐Dnmt protein. (A,B) T293 cells were infected with ZF‐Dnmt1C, ZFDnmt3aC, ZF‐Dnmt3a‐1C or Ad‐control, and the expression of recombinant proteins was checked at 48 h.

To study the expression of ZF‐Dnmts from Ad‐ZF‐Dnmt vectors, T293 cells were infected with Ad‐ZF‐Dnmts or Ad‐control. The recombinant ZF‐Dnmt3aC, Ad‐ZF‐Dnmt1C, and ZF‐Dnmt3aC‐1C were successfully expressed in T293 cells as shown in Fig. 2.

### Recombinant ZF‐Dnmt3aC‐1C suppresses PD‐L1 expression more efficiently

**Figure 3:**
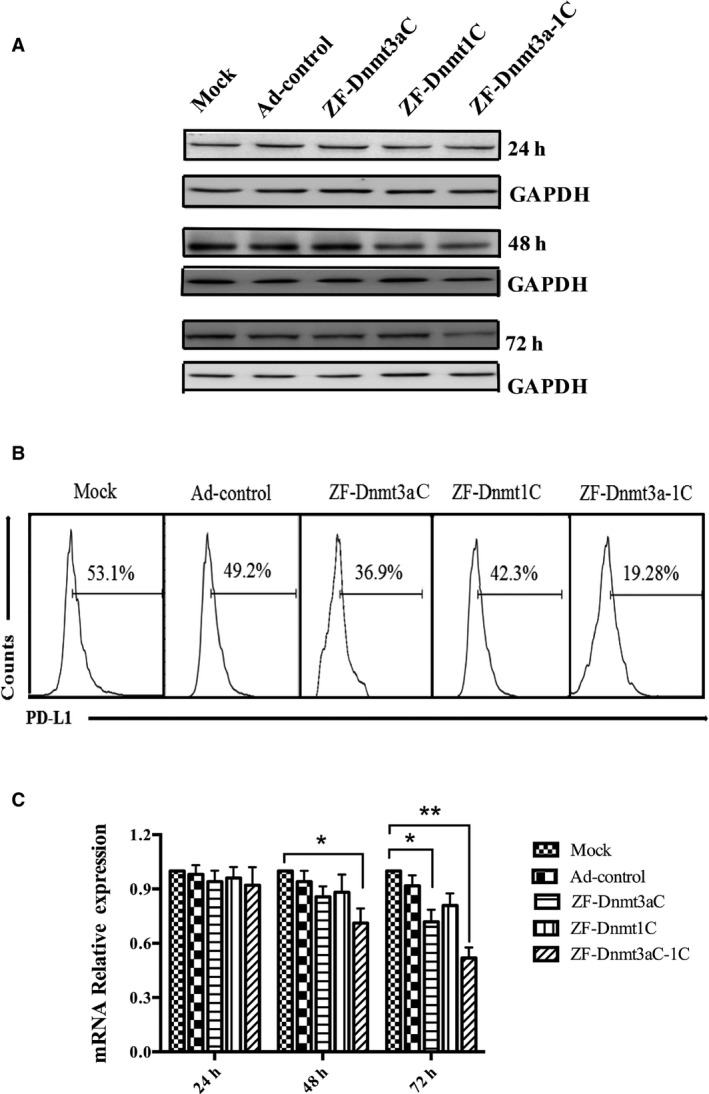
Effect of ZF‐Dnmt3aC‐1C on the expression of PD‐L1. Human prostate cancer cells (DU145) were transduced with Ad‐ZF‐Dnmts or Ad‐control, and the expression of PD‐L1 was checked at 24, 48, and 72 h. (A) Western blot analysis, (B) flow cytometry, (C) qRT–PCR. Error bars represent ± SD.(*P <* *0.05, **P < 0.01).

Human prostate cancer cells (DU145) were transduced with Ad‐ZF‐Dnmt3aC, Ad‐ZF‐Dnmt3aC‐1C, or Ad‐control, and the expression of PD‐L1 was checked at 24, 48, and 72 h. Western blot analysis showed that ZF‐Dnmt3aC‐1C suppressed the expression of PD‐L1 more efficiently as compared to ZF‐Dnmt3aC alone and the expression level was reduced to less than half after 72 h as shown in Fig. 3A. This was further authenticated by flow cytometry results. Flow cytometry analysis showed that as compared to the mock (53.1%) and Ad‐control group (49.2%), ZF‐Dnmt3aC‐1C suppressed the expression of PD‐L1 to 19.28% (Fig. 3B). These results were further confirmed at the level of mRNA expression as assessed by qRT–PCR at the different time intervals showing that the expression of PD‐L1 was reduced by 50–60% by ZF‐Dnmt3aC‐1C (Fig. 3C). These results show that the addition of Dnmt1C to the Dnmt3aC improves its biological activity of methylation and hence suppresses the expression of PD‐L1 more efficiently.

### Degree of methylation by Methylation‐Specific PCR (MSP) assay

**Figure 4:**
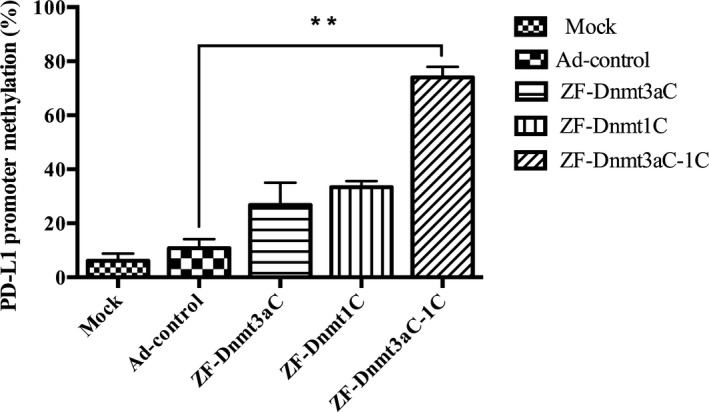
Methylation‐specific PCR assay: MSP assay was performed 72 h after cotransfection of DU145 with Ad‐control and Ad‐ZF‐Dnmts. Error bars represent ± SD.(**P <* *0.01) .

Methylation‐specific PCR assay was performed 72 h after transfection of DU145 cells with Ad‐control or Ad‐ZF‐Dnmts. The result showed lower methylation for mock (16%) and control group (18.7%) than the experimental groups. The Ad‐ZF‐Dnmt3aC showed 25.7%, Ad‐ZF‐Dnmt1C showed 35.3%, and Ad‐ZF‐Dnmt3aC‐1C group showed highest methylation reaching 84%. **(**Fig. 4). These results further support the idea that fusion of Dnmt1C to the Dnmt3aC improves its biological activity of methylation.

## Discussion

Targeting the DNA methylation‐based gene‐silencing machinery has assumed great significance in the field of cancer epigenomics 15.We applied this idea to the suppression of PD‐L1 gene expression to block PD‐1/PD‐L1 interaction. First, we identified the potential methylation sites and zinc finger binding site in the promoter region of PD‐L1 gene. Then, we constructed recombinant plasmids consisting of the C‐terminal domains of both Dnmt1 and Dnmt3a methyltransferases fused to a zinc finger domain that specifically bind to PD‐L1 promoter of prostate cancer cell line DU145. Methylation‐specific PCR showed that the designed constructs have successfully methylated the promoter sequences in the experimental group. Further results showed that treatment of DU145 cells with this protein significantly reduced the expression of PD‐L1 as evaluated at antibody‐specific western blot. This was further supported by the result of flow cytometry, which showed that mock and control group is 53.1% and 49.2%, respectively, while the experimental group decreased to 19.28%. However, the mock group which was treated with the empty vectors did not show such reduction in the expression of PD‐L1 level. As a whole, the promoter sequence of PD‐L1 was methylated which suppressed the expression level of PD‐L1.

Gevensleben *et al*. reported that there is an inverse correlation between PD‐L1* *promoter methylation and protein expression in prostate cancer. They suggest that PD‐L1 is inversely correlated with miR as cellular component, which modifies the downstream processing of PD‐L1 mRNA. Differential expression of miR may therefore potentially interfere with the linear translation of PD‐L1 mRNA into PD‐L1 protein 16. D. Goltz *et al*. also report on the inverse correlation between promoter methylation and respective mRNA expression 17, 18. These studies support our results of suppression of PD‐L1 by methylating its promoter.

Further we evaluated the cooperative role of different Dnmts with respect to DNA methylation and subsequent PD‐L1 expression. The specific roles of these Dnmts in *de novo* and maintenance functions have recently been re‐examined. Although single knockout of either Dnmt1 or Dnmt3b gene had minimal effects on DNA demethylation in colon cancer cells, 19 knockouts of both Dnmt1 and Dnmt3b genes led to demethylation and re‐expression of tumor suppressor genes in these cells 20. This observation implies that Dnmt1 and Dnmt3b together cooperate to maintain DNA methylation patterns and to silence genes in human cancer cells. In separate knockdown studies using RNA interference techniques, double RNA interference of Dnmt1 plus Dnmt3b enhanced DNA demethylation and gene reactivation 21. El‐Osta reported that the methyltransferases Dnmt1 and Dnmt3b cooperatively maintain DNA methylation and gene silencing in human cancer cells. Disruption of the human Dnmt3b only slightly reduces the overall global DNA methylation; however, demethylation was markedly potentiated when both Dnmt1 and Dnmt3b were simultaneously deleted 22. Based on this, we hypothesized that the cooperative role of Dnmt3a and Dnmt1 might augment the biological function of Dnmt3a. We fused the C‐terminal of Dnmt1 (Dnmt1C) to the Dnmmt3aC and evaluated the expression of PD‐L1. Our results showed that the addition of Dnmt1C to Dnmt3aC synergize its biological function and improved its methylation efficiency. Thus Dnmt3aC‐1C suppressed PD‐L1 expression more efficiently as compared to Dnnmt3ac alone. This concept of combining Dnmts can be extended to suppress other genes.

## Conflict of interest

The authors declare no conflict of interest.

## Author contributions

NZ, JH, XL, and ZW conceived and designed the project. XL and ZW performed the experimental work. XL wrote the manuscript, researched the data, reviewed and edited the manuscript. JX, HL, SZ, HY, LZ, BH, LY, and LL contributed to the discussion. Funding acquisition by NZ.
